# On the Evolutionary and Biogeographic History of *Saxifraga* sect. *Trachyphyllum* (Gaud.) Koch (Saxifragaceae Juss.)

**DOI:** 10.1371/journal.pone.0069814

**Published:** 2013-07-26

**Authors:** Eric G. DeChaine, Stacy A. Anderson, Jennifer M. McNew, Barry M. Wendling

**Affiliations:** Department of Biology, Western Washington University, Bellingham, Washington, United States of America; CNRS/Université Joseph-Fourier, France

## Abstract

Arctic-alpine plants in the genus *Saxifraga* L. (Saxifragaceae Juss.) provide an excellent system for investigating the process of diversification in northern regions. Yet, sect. *Trachyphyllum* (Gaud.) Koch, which is comprised of about 8 to 26 species, has still not been explored by molecular systematists even though taxonomists concur that the section needs to be thoroughly re-examined. Our goals were to use chloroplast *trn*L-F and nuclear ITS DNA sequence data to circumscribe the section phylogenetically, test models of geographically-based population divergence, and assess the utility of morphological characters in estimating evolutionary relationships. To do so, we sequenced both genetic markers for 19 taxa within the section. The phylogenetic inferences of sect. *Trachyphyllum* using maximum likelihood and Bayesian analyses showed that the section is polyphyletic, with *S. aspera* L. and *S bryoides* L. falling outside the main clade. In addition, the analyses supported several taxonomic re-classifications to prior names. We used two approaches to test biogeographic hypotheses: i) a coalescent approach in Mesquite to test the fit of our reconstructed gene trees to geographically-based models of population divergence and ii) a maximum likelihood inference in Lagrange. These tests uncovered strong support for an origin of the clade in the Southern Rocky Mountains of North America followed by dispersal and divergence episodes across refugia. Finally we adopted a stochastic character mapping approach in SIMMAP to investigate the utility of morphological characters in estimating evolutionary relationships among taxa. We found that few morphological characters were phylogenetically informative and many were misleading. Our molecular analyses provide a foundation for the diversity and evolutionary relationships within sect. *Trachyphyllum* and hypotheses for better understanding the patterns and processes of divergence in this section, other saxifrages, and plants inhabiting the North Pacific Rim.

## Introduction

Plants of the genus *Saxifraga* L. (Saxifragaceae Juss.) have been used extensively in the fields of systematics and phylogeography to broaden our understanding of the patterns and processes of diversification in arctic and alpine regions. Because *Saxifraga* has a primarily arctic-alpine distribution and those regions are dramatically impacted by climatic variability [1], saxifrages are excellent organisms for investigating biotic responses to climate change. Studies on a suite of saxifrages have helped to develop the roles that geographic isolation and dispersal have played in the process of speciation [2], [3], [4], [5]. Indeed, in depth studies concerning the evolutionary history of *S. oppositifolia* L. [6], [7], [8] serve as a foundation for how arctic-alpine plants have responded to the climatic shifts of the Quaternary. Phylogenies form the basis of such studies, and have been generated for the Saxifragaceae [9], *Saxifraga* [10], and the major sections within *Saxifraga*, including sect. *Ciliatae* Haworth [11], sect. *Ligulatae* Haworth [12], and sect. *Saxifraga* Webb & Gornall [13]. Yet, sect. *Trachyphyllum* (Gaud.) Koch has been largely ignored by molecular systematists even though the section resides in a basal position within the genus and thus likely holds valuable clues concerning the origin and evolutionary history of *Saxifraga* and other flowering plants of arctic-alpine regions. Moreover, taxonomists agree that a thorough examination of this section is needed [14], [15], [16], [17], [18].

As currently defined, sect. *Trachyphyllum* consists of anywhere from approximately 8–26 species (Table 1), depending on the taxonomic treatment, with Europeans and North Americans [15], [17], [19] accepting few species and Russian botanists adopting the more narrow species concept with many accepted species [14], [20], [21], [22]. Gaudin [23] first described sect. *Trachyphyllum* as being herbaceous with lanceolate, spinulose-ciliate leaves and white flowers and assigned *S. aspera* L. as the type for the section. As the common name ‘rough-leaved saxifrages’ implies, the stiff hairs along the margins and tips of their evergreen leaves give them a rough appearance and feel. The constituent taxa are perennials that grow in loose to dense mats. Their leaves are alternate, narrow, usually lanceolate, stiff, either entire or 3-lobed at the tip, and arranged in tight rosettes. While the margins typically exhibit strong, broad-based hairs, the tip may be apiculate or strongly mucronate. Both leaf surfaces are generally hairless and lime-secreting hydathodes are absent. Glandular hairs are multiseriate. Axillary buds are prominent but not summer dormant. The flowering stems are leafy and terminal with flowers that are usually regular. The sepals are not reflexed. Petals are white to pale yellow, usually with spots (yellow, orange, red, or purple) or a deep yellow patch at the base. The ovary is superior or nearly so. The most distinguishing characteristics for separating species in the section are leaf shape and size, length of marginal hairs, and the shape, color, and spotting on the petals [16], though some of these traits (e.g., leaves) are correlated with habitat [15], [19] and others (petal color and spotting) may be lost in herbaria specimens [19].

**Table 1:** Current taxonomy, distribution, and sampling of *Saxifraga* sect. *Trachyphyllum*.

Current nomenclature 1	Suggestions 2	Distribution 3	N	Herbaria accession	GenBank accession
*S. algisii* Egorova & Sipliv.^4^		RU: Siberia	0	-	
*S. arinae* Zhmylev^4^		RU: Shikotan Island	0	-	
*S. aspera* L.		EU: Pyrenees, Alps, Apennines	2	RBGE-E00421806	KF196319;
				WWB-22810	KF196371-72
*S. balandinii* Zhmylev^4^		RU: Kamchatka	0	-	
*S. bronchialis* L.		RU: northern RU to Pacific Coast	2	WTU-283068, 282875	KF196360-61; KF196396-97
*S. bronchialis* L. subsp. *anadyrensis* (Losinsk.) Kozhern		RU: Magadan to mouth of Lena River	2	ALA-V154288, V78524	KF196358; KF196374
*S. bronchialis L.* subsp. *austromontana* (Wiegand) Piper	*S. austromontana* Wiegand	NA: Cascade Mtns. , Rocky Mtns.	11	RMH-619568, 750642, 750643, 750647, 758235,	KF196332-38, KF196343-47;
				779011, 780148	KF196398-409
				WTU-361429, 369601	
				WWB-22800, 22801	
*S. bronchialis L.* subsp. *codyana* (Zhmylev) Cody	*S. codyana* Zhmylev	NA: Alaska, Yukon	1	UBC-V212762	KF196327; KF196379
*S. bronchialis L.* subsp. *funstonii* (Small) Hultén	*S. funstonii* (Small) Fedde	RU: Taimyr to Chukotka, Kamchatka;	3	ALA-V121070, V156966, V156968	KF196326,28,29-31,62,65;
		NA: Alaska, Yukon, British Columbia	4	WTU-356796	KF196393-95,
				WWB-22807, 22808, 22809	KF196410-12,17
*S. bryoides* L.		EU: Pyrenees, Alps, Carpathians	1	WWB-22811	KF196318; KF196373
*S. caulescens* Sipliv.		RU: Siberia; Mongolia	1	RBGE-E00258082	KF196325; KF196375
*S. cherlerioides* D. Don		RU: Sakhalin, Kuril Islands, OkhotskNA: Alaska, Yukon	3	WTU-358172, 358184, 358185	KF196363-64, KF196366; KF196376-78
*S. cherlerioides* D. Don subsp. *ascoldica* (Sipliv.) Vorosch.^4^		RU: Siberia	0	-	
*S. derbekii* Sipliv.		RU: Far East near Sea of Okhotsk	1	UBC-V164312	KF196367; KF196380
*S. kolymensis* A. P. Khokhr.^4^		RU: Magadan	0	-	
*S. kruhsiana* Fisch. ex Ser.		RU: Kamchatka, Okhotsk	2	ALA-V10779, V10779	KF196356-57; KF196381-82
*S. nishidae* Miyabe & Kudô		Japan: Hokkaido: Yuubari Mountains	1	RBGE-E00295524	KF196324; KF196383
*S. omolojensis* A. P. Khokhr.		RU: Chukotka to Okhotsk	1	ALA-V129124	KF196368; KF196384
*S. rebunshirensis* (Engl. & Irmsch.) Sipliv.		Japan: Rebun; RU: Sakhalin	1	UBC-V164570	KF196323; KF196385
*S. spinulosa* Adams		RU: Urals to Chukotka, Kamchatka	2	ALA-V107682, V168369	KF196354-55; KF196386-87
*S. stelleriana* Merk. ex Ser.	*S. bronchialis* L.	RU: Angara to Okhotsk, Zea, Lk. Baikal	1	ALA-V154286	KF196359; KF196388
*S. taylorii* Calder & Savile		NA: British Columbia - Haida Gwaii	3	UBC-V214354, V214965, WWB-22805	KF196351-53; KF196389-90
*S. tricuspidata* Rottb.		NA: Alaska, Canada, Greenland	4	RMH-363418	KF196339-42;
				WWB-22802, 22803, 22804	KF196413-16
*S. vespertina* (Small) Fedde		NA: Olympic Mtns., Cascade Mtns.	3	WTU-284404, UBC-V159757, WWB-22806	KF196348-50; KF196391-92

N is the number of individuals sequenced per taxon.

1 Taxon names were confirmed through IPNI, Tropicos, and The Plant List.

2 Suggested taxonomic revisions based on our phylogenetic inferences.

3 Abbreviations used for geographic locations: EU = Europe, NA = North America, RU = Russia.

4 Unresolved taxon of sect. *Trachyphyllum* not available for this study.

Since the earliest descriptions, members of the section have languished in a confusing taxonomic mire (see Table 2 for nomenclatural history), in part because of the use of environmentally-impacted characters in describing species, the reliance on herbaria rather than fresh specimens, and also due to the aforementioned disjunct distribution and potential limited communication of taxonomists with different systematic philosophies. For the most part, species in the section are clearly, morphologically distinct and their status as valid species is not in question. However, that is not the case for *S. bronchialis* L. and morphologically similar species, subspecies, and varieties, which constitute at least half of the taxonomic diversity in sect. *Trachyphyllum*. The confusion apparently began when Linnaeus described *S. bronchialis* in 1753 [24]. Linnaeus labeled the only specimen of *S. bronchialis* that he ever saw as *S. aspera*. Subsequently, he decided to make *S. bronchialis* a separate species but potentially forgot to change his annotation [15]. This was further complicated because most Russian botanists would now assign that specimen to *S. spinulosa* Adams, based on geographic location. Currently, many taxonomists attribute much of the diversity in this section to subspecies, varieties, and geographic races of a nearly circumpolar *S. bronchialis*. Still, others denote only three subspecies of *S. bronchialis* - one Siberian/European, one Beringian, and one in the Rocky Mountains and Cascades of North America - with the other taxa being given the status of species (Table 1).

**Table 2:** Taxonomic history of the circumscription of *Saxifraga* sect. *Trachyphyllum*.

Author	Date	Genus	Section	Sub-section	Taxa included 1	Ref
Linnaeus	1753	*Saxifraga*			*S. aspera, S. bronchialis, S. bryoides*	[24]
Haworth	1821	*Ciliaria*			*S. aspera, S. bronchialis, S. bryoides*	[70]
		*Leptasea*			*S. tricuspidata*	
Don	1822	*Saxifraga*	*Leiogyne*		*S. aspera, S. bronchialis, S. bryoides, S. cherlerioides*	[71]
			*Verae*		*S. tricuspidata*	
Gaudin		*Saxifraga*	*Trachyphyllum*		*S. aspera, S. bryoides*	[23]
Koch	1836	*Saxifraga*	*Trachyphyllum*		*S. aspera, S. bryoides, S. tenella* Wulfen, *S. aizoides* L.	[94]
Small	1905	*Leptasea*			*S. aspera, S. bronchialis, S. bronchialis* subsp. *austromontana* 2, *S. bronchialis* subsp. *funstonii* 2, *S. bryoides, S. cherlerioides, S. tricuspidata, S. vespertina*	[57]
Engler & Irmscher	1919	*Saxifraga*	*Trachyphyllum*		*S. aspera, S. bronchialis, S. bronchialis* subsp. *austromontana* 3, *S. bronchialis* subsp. *funstonii* 3, *S. bryoides* 3, *S. cherlerioides* 3, *S. kruhsiana* 3, *S. omolojensis* 4, *S. rebunshirensis* 3, *S. spinulosa* 3, *S. stelleriana* 3, *S. tricuspidata, S. vespertina* 3	[58]
Losina-Losinskaya^5^	1939	*Saxifraga*	*Trachyphyllum*		*S. bronchialis, S. bronchialis* subsp. *anadyrensis, S. cherlerioides, S. spinulosa, S. eschscholtzii* Sternb.	[25]
Calder & Savile^6^	1957	*Saxifraga*	*Trachyphyllum*		*S. bronchialis* subsp. *austromontana, S. bronchialis* subsp. *funstonii, S. cherlerioides, S. taylorii, S. tricuspidata, S. vespertina*	[19]
Siplivinsky^7^	1971	*Saxifraga*	*Trachyphyllum*		*S. algisii, S. ascoldica, S. bronchialis, S. bronchialis* subsp. *anadyrensis, S. bronchialis* subsp. *funstonii, S. caulescens, S. cherlerioides, S. derbekii, S. kruhsiana, S. omolojensis* 4, *S. rebunshirensis, S. spinulosa, S. stelleriana*	[20]
Khokhryakov^7^	1979	*Saxifraga*	*Trachyphyllum*	*Criomorphicae*	*S. algisii, S. bronchialis* subsp. *funstonii, S. cherlerioides, S. kruhsiana, S. spinulosa*	[21]
				*Xeromorphicae*	*S. ascoldica, S. bronchialis, S. bronchialis* subsp. *anadyrensis, S. caulescens, S. derbekii, S. kolymensis, S. omolojensis, S. rebunshirensis*	
Zhmylev & Khokhryakov	1985	*Saxifraga*	*Trachyphyllum*	*Criomorphicae*	Same as Khokhryakov, excluding *S. algisii* but including *S. bronchialis* subsp. *austromontana, S. vespertina*	[22]
				*Xeromorphicae*	Same as Khokhryakov, but also *S. algisii*	
				*Dentaphyllae*	*S. tricuspidata, S. taylorii*	
				*Fibrosophyllae*	*S. aspera, S. bryoides*	
Weber	1982	*Ciliaria*			*S. bronchialis* subsp. *austromontana, S. bronchialis* subsp. *funstonii, S. tricuspidata, S. vespertina*	[95]
Gornall	1987	*Saxifraga*	*Trachyphyllum*		Same as Engler & Irmscher, but also *S. algisii, S. ascoldica, S. bronchialis* subsp. *anadyrensis, S. caulescens, S. derbekii, S. kolymensis, S. taylorii*	[59]
McGregor	2008	*Saxifraga*	*Trachyphyllum*		Same as Gornall, but also *S. balandinii*, *S. nishidae*	[16]

1 The list of included species uses currently accepted names (see Table 1) and is only exhaustive for *S.* sect. *Trachyphyllum*. For the other genera, the lists of taxa are abbreviated to only show species circumscribed at some point within *S.* sect. *Trachyphyllum*.

2 Small considered these taxa individual species.

3 Engler and Irmscher considered *S. bryoides* a subspecies of *S. aspera* and the others as varieties within *S. bronchialis*.

4 Given as *S. multiflora* in these references.

5 Losina-Losinskaya only focused on taxa occurring within the U.S.S.R. and assumed *S. bronchialis* subsp. *funstonii* synonymous with *S. cherlerioides*.

6 Calder and Savile only focused on North American taxa.

7 Siplivinsky and Khokhryakov were only concerned with taxa of the U.S.S.R.

In an attempt to categorize the variation and better delineate taxa, especially those similar to *S. bronchialis*, sect. *Trachyphyllum* was divided into subsections (Table 2) and series based on a few ‘discerning’ characters. Khokhryakov [21] was the first to attempt an intra-section classification scheme, but focused only on the northeast Asian taxa, dividing them into the *Xeromorphicae* A. P. Khokhr. and *Criomorphicae* A. P. Khokhr., with three and two series respectively. The two subsections were distinguished by i) rigid, straight, linear, needle-like, pointed leaves, white petals, with yellow and red dots vs. ii) curved, non-rigid, broad leaves with white or yellow petals, and apunctate. Subsequently, Zhmylev and Khokhryakov [22] re-examined the sub-sectional classification for the entire sect. *Trachyphyllum*, adding those from Europe and North America. *Fibrosophyllae* Zhmylev & A. P. Khokhr. (*S. aspera* and *S. bryoides* L.) were noted for having soft, gray-green, lanceolate, leaves and spherical capsules and because this section was the most isolated both morphologically and geographically, the authors suggested that it may warrant the rank of an independent section. The fourth subsection, *Dentaphyllae* Zhmylev & A. P. Khokhr., including *S. tricuspidata* Rottb. and *S. vespertina* (Small) Fedde, was clearly differentiated from the others by 3-toothed leaves. More recently, two of the subsections (*Xeromorphicae* and *Criomorphicae*) have been discussed in terms of the Bronchialis and Spinulosa ‘species aggregates or complexes,’ respectively [15], [16], [17], [18].

**Figure 1:**
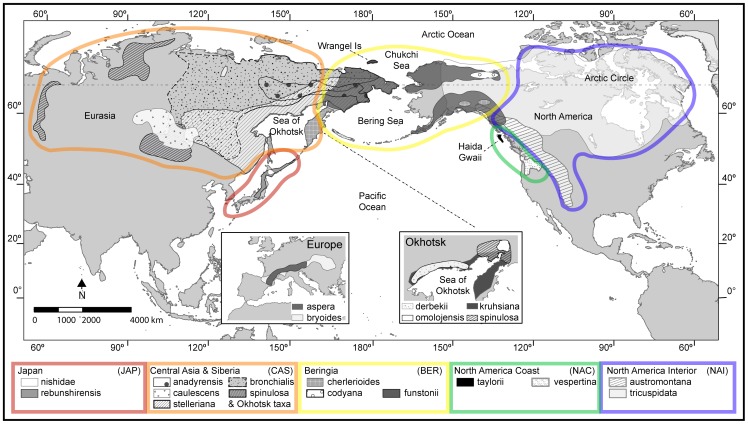
Distribution map of *Saxifraga* sect. *Trachyphyllum*. Distributions for all taxa are approximate based on herbaria records and literature descriptions [14], [16], [19], [20], [58]. All taxa used in the study are shown and labeled with their specific or subspecific epithet. The major regions discussed in the text and investigated through biogeographic analyses are outlined in color: North America Interior (NAI) = blue; North America Coast (NAC) = green; Beringia (BER) = yellow; Central Asia & Siberia (CAS) = orange; Japan (JAP) = red. An inset map is provided for *S. aspera* and *S. bryoides* inhabiting the mountains of Europe because the ranges for those taxa are disjunct from the others. For greater resolution on the distributions of endemic taxa around the Sea of Okhotsk, the inset map for that region excludes *S. bronchialis*, *S. bronchialis* subsp. *anadyrensis*, *S. cherlerioides*, *S. funstonii*, and *S. stelleriana*. The Miller cylindrical projection map was generated using ArcMap [93].

The section is found in the arctic-alpine and subalpine environments, with a primarily trans-Beringian distribution though two species are restricted to the mountains of Europe (Table 1, Fig. 1). The majority of the taxa are patchily and narrowly distributed in a geographic arc around the north Pacific. In contrast, *S. bronchialis* s. str. is widespread across that entire range. In the North American interior (NAI) *S. bronchialis* subsp. *austromontana* (Wiegand) Piper inhabits the alpine and subalpine of the Rocky Mountains, Cascades, and Olympics, while *S. tricuspidata* occurs mainly in the north from Alaska to Newfoundland and south into the mountains of British Columbia, and also in Greenland. *Saxifraga vespertina* and *S. taylorii* Calder & Savile (endemic to Haida Gwaii) are restricted to the North American coast (NAC) in the Pacific Northwest. Beringia (BER), including Alaska, northwestern Canada, eastern Siberia, and northern islands such as Wrangel Island, is home to *S. bronchialis* subsp. *funstonii* (Small) Hultén, *S. bronchialis* subsp. *codyana* (Zhmylev) Cody, and *S. cherlerioides* D. Don. Many species have been described from central Asia and Siberia (CAS), from Chukotka to Baikal and China and south to Kamchatka, including *S. caulescens* Sipliv., *S. bronchialis*, *S. spinulosa* and their putative relatives. Japan and southern, coastal Asia (JAP) harbors a limited number of endemic taxa: *S. nishidae* Miyabe & Kudô and *S. rebunshirensis* (Engl. & Irmsch.) Sipliv. Interestingly, *S. aspera* and *S. bryoides* are confined to the European mountains and are seen as being an extension from the more typical trans-Beringian distribution [15]. The relatively high degree of endemism within this section and the potential for geographic structure suggests that isolation in multiple refugia may have been a driving force in the diversification of sect. *Trachyphyllum* [19], [25].

**Figure 2:**
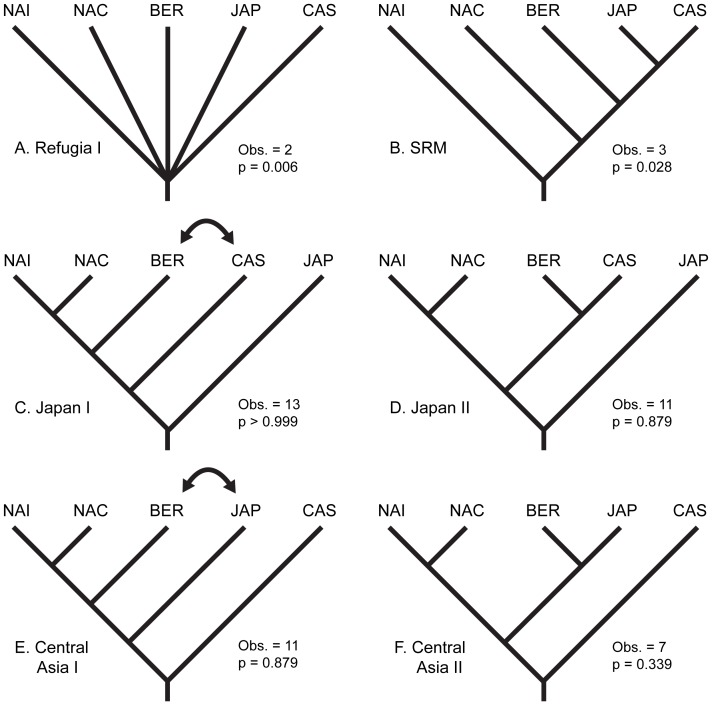
Geographic models of population divergence. Models of population divergence based on the location of potential glacial refugia and possible origins and migration routes are shown as follows: A) Refugia - fragmentation of an ancestral population among all refugia, B) Southern Rocky Mountain (SRM) - an origin in the southern Rocky Mountains of North America followed by westward dispersal, C) and D) Japan I and II - origin in Japan with northern and eastward dispersal, and E) and F) Central Asia I and II - an Asian origin with southern and eastward dispersal. NAI = North America Interior, NAC = North America Cascadia, BER = Beringia, CAS = Central Asia & Siberia, JAP = Japan. Observed DC values and P-values for each model are shown. The coalescent simulations produced a distribution of DC values with mean = 8.18 and st. dev. = 2.34. The Refugia (A) and the SRM (B) models were supported at α<0.05. All others were rejected.

The main goal of this study was to resolve the evolutionary history of sect. *Trachyphyllum* using molecular data. To do so, we i) circumscribed the section by inferring the phylogenetic position of potential taxa within the broad *Saxifraga* tree using internal transcribed spacers (ITS) of nuclear ribosomal DNA and chloroplast *trn*L-F and markers. The ITS and the *trn*L-F regions have been the most widely used markers for inferring evolutionary relationships within *Saxifraga* [10], [11], [12], [13] because they yield relatively good resolution at the species level [13], [26]. Thus, these regions provide the most previously sequenced taxa for the best means of placing sect. *Trachyphyllum* within the *Saxifraga* tree. These phylogenetic analyses allowed us to ii) identify sub-clades within sect. *Trachyphyllum*, iii) test geographically-based diversification hypotheses by performing coalescent simulations in Mesquite 2.75 [27] (Fig. 2) and maximum likelihood tests of geographic range evolution in Lagrange [28], and iv) investigate the utility of morphological characters in resolving sub-sectional classifications using a stochastic character mapping approach in SIMMAP 1.5 [29]. This is the first molecular examination of sect. *Trachyphyllum* and includes the vast majority of the putative species.

## Materials and Methods

### Specimen sampling

Specimens of sect. *Trachyphyllum* used in the genetic analyses were either collected directly from the field or obtained through herbaria loans (see Table 1 for accession numbers). Freshly collected samples were stored on ice or dried on silica gel prior to storage at −20°C. For the newly collected specimens, collecting permits were provided by the Arctic National Wildlife Refuge (permit #2006-S6), the Gates of the Arctic National Park (GAAR-2008-SCI-0001), and the Noatak National Preserve (NOAT-2008-SCI-0003) and voucher specimens were archived at the Western Washington University Herbarium (WWB). We sequenced specimens of 19 taxa (Table 1), including at least one individual of all the generally accepted species as well as many additional taxa of interest. The number of individuals sampled from the five regions (Fig. 1) used in the geographic analyses are as follows: NAI = 15, NAC = 6, BER = 11, CAS = 11, JAP = 3. Given their extensive and disjunct distributions, questionable taxonomy, and greater accessibility, we included more specimens of *S. bronchialis* subsp. *austromontana* and *S. bronchialis* subsp. *funstonii* in the analyses than the other taxa. On the other hand, most members of sect. *Trachyphyllum* have very restricted geographic distributions (Fig. 1), which made obtaining samples difficult.

In order to circumscribe sect. *Trachyphyllum* and place the section within the broader *Saxifraga* phylogeny, we also obtained sequence data for other sections within *Saxifraga* and used additional Saxifragaceae as outgroups following Soltis et al. [9]. For the vast majority of specimens (99 taxa), data was downloaded from GenBank (see Appendix S1 for accession numbers). To this dataset, we added new DNA sequence data for *Micranthes hieraciifolia* (Waldstein & Kitaibel ex Willdenow) Haworth, *M. ferruginea* (Graham) Brouillet & Gornall, *Saxifraga oppositifolia*, *S. flagellaris* Wildenow, and *S. eschscholtzii* Sternb.

### DNA extraction and phylogenetic reconstructions

For all newly generated sequence data, total genomic DNA was isolated from leaf tissue using the MasterPure DNA Leaf Extraction Kit (Epicentre). Both molecular markers were PCR amplified following published protocols. We used primers trnLb2(UAA) and trnF(GAA) for the *trn*L-F region [30]. The entire ITS1, 5.8S and ITS2 region was amplified using primers 17SE and 26SE [31]. The *trn*L-F PCR products were sequenced directly, but ITS products were cloned using TOPO TA cloning kits (Invitrogen, Carlsbad, California) prior to sequencing. Purified plasmids from clones or PCR products were sequenced in both directions by the University of Washington High Throughput Genomics Unit (Seattle, WA). Sequences were then manually edited in Sequencher v4.8 (Gene Codes Corp.) and archived in GenBank (Table 1).

We performed additional quality control on all the ITS sequences because the region has known phylogenetic concerns [32]: i) evolving via concerted evolution, potentially obscuring true species phylogenies [33], [34], ii) potential mistakes derived from comparing paralogous copies [35], [36], iii) and potentially misleading inferences at higher levels [37]. To overcome these, we followed the guidelines of Feliner and Rosselló [38]: i) cloned all sequences, ii) partitioned each sequence into ITS1, 5.8S, and ITS2, iii) determined if the sequence was of fungal origin by performing a BLAST search against the NCBI fungal database, iv) examined the secondary structure of each region using GeneBee (www.genebee.msu.su/services/rna2_reduced.html) and the ITS2 Database [39] for expected structures, and v) examined each of the partitioned sequences for expected motifs and length. Furthermore, the inferred secondary structures were used in sequence alignments. Finally, models of DNA substitution were estimated for each region independently and datasets were partitioned in the phylogenetic analyses (described below).

Both the *trn*L-F and ITS sequence datasets were aligned in ClustalX 2.0 [40] and manually checked in MacClade 4.08a [41]. Sites of ambiguous homology and INDELS were excluded from analyses. Models of nucleotide substitution for all molecular markers were estimated in jModeltest 0.1.1 [42] as follows: *trn*L-F TVM+I+ Γ, ITS1 TIM2ef+I, 5.8S TIM3ef+I+ Γ, ITS2 TIM3ef+ Γ). Three independent sets of phylogenetic analyses were performed: i) *Saxifraga* based on the entire ITS dataset, ii) *Saxifraga* based on the entire *trn*L-F dataset, and iii) sect. *Trachyphyllum* based solely on a taxonomically reduced ITS dataset focused on members of the section and using only sequences generated in this study, such that there were no missing data for any individuals in the analysis. We analyzed the *trn*L-F and ITS markers separately because organelle and nuclear DNA have likely evolved under different population histories [43], [44]. For each of the three analyses, we used maximum likelihood (ML) and Bayesian methods to reconstruct phylogenies. All trees were visualized in FigTree 1.3.1 [45] and archived in TreeBASE (http://purl.org/phylo/treebase/phylows/study/TB2:S14345). The large discrepancy between the taxonomic datasets for the *trn*L-F and ITS analyses precluded formal tests of congruence between the trees.

Garli 2.0 [46] was employed to reconstruct the ML trees. Optimal ML trees for the *trn*L-F dataset were identified in Garli using the DNA substitution model estimated in jModeltest, with ten replicates per analysis. For the ITS datasets, the three regions (ITS1, 5.8S, ITS2) were partitioned with the appropriate model applied to each partition. For all the ML analyses, nodal support was assessed using 200 bootstrap replicates with 5 analysis replicates per bootstrap.

Bayesian phylogenetic analyses were performed in MrBayes 3.2 [47]. For the ITS datasets, models of DNA substitution and rates of evolution were allowed to vary among partitions. All analyses were run for 4 million generations, with two simultaneous analyses, four chains each, using the default priors and repeated 3 times from different random seeds to confirm convergence on similar topologies. Twenty-five percent of the trees were discarded as burn-in based on stationarity as determined by both the measure of similarity of tree samples calculated in MrBayes (with average standard deviation of split frequencies <0.01) and by visualizing posterior distributions in Tracer v1.5 [48].

### Tests of biogeographic history for sect. *Trachyphyllum*

First, we tested geographic models of population divergence through coalescent simulations of gene trees in a fashion similar to that pioneered by Knowles [49] that gave rise to the field of statistical phylogeography [50]. Mesquite 2.75 [27] was employed to test four general geographic models of population divergence visualized as species trees (Fig. 2). This is a conservative approach that does not rely on estimates of effective population size (Ne) or divergence time, but provides statistical tests that support or refute simple biogeographic hypotheses. Models of population divergence were created based on the geographic distribution of hypothesized refugial areas, with each region as the possible origin and potential routes of dispersal: i) Refugia - fragmentation of an ancestral population among all refugia, ii) Southern Rocky Mountain (SRM) - an origin in the southern Rocky Mountains of North America followed by westward dispersal and divergence across Beringia and then south and further west in Asia, iii) Japan - origin in Japan with eastward dispersal and divergence across Beringia then south and further east in North America, and iv) Central Asia - and Asian origin with southern and eastward dispersal and divergence across Beringia then south and further east in North America. We tested whether the fit of the observed gene tree (reconstructed through our bayesian phylogenetic analyses of sect. *Trachyphyllum* using the ITS data) to each model of population divergence was better than expected by random. First we estimated the amount of discordance between our observed tree and each model (Fig. 2) using the deep coalescence (DC [51]), which assumes discordance is due to incomplete lineage sorting. Then, we generated a null distribution of DC values based on coalescent simulations [49], [52], [53]. To do so, we used our observed gene tree as the ‘constrained’ tree to simulate 10,000 ‘constraining’ trees (divergence models, which can be thought of as species trees) that could have given rise to the observed tree. The DC for each simulated constraining tree was then estimated by comparing it to the observed tree to yield a null frequency distribution of 10,000 DC values. The DC value for the observed sect. *Trachyphyllum* tree was compared with the simulated distribution to statistically test whether the observed tree was more likely to have arisen under the given model than by chance. If the DC value for the observed tree constrained within a given model fell within that of the null distribution (at p >0.05), that model was rejected. If, however, the DC value for the observed tree constrained within a given model gave a better fit than expected by the null distribution (at p<0.05), then the model was accepted as a possible scenario that could have led to the distribution of the genetically distinct taxa observed today.

Next, we used Lagrange [28], [54] to test more complex models of directional biogeographic expansion and infer the geographic origin of the clade. Lagrange v. 20120508 [28] employs a maximum likelihood framework for testing models of geographic range evolution based on a dispersal-extinction-cladogenesis model. To maintain consistency between our approaches, we used our bayesian phylogeny reconstructed from the ITS data as our tree, but modified it to meet the requirements of the analyses: we scaled its length to absolute time (220 ky) based on a 1.72 substitutions/site/year rate estimated for the ITS in *Saxifraga* (an estimate that fits well with other rates for herbaceous angiosperms [55]), and made all nodes bifurcating. We constructed five models, similar to but more complex than those used in the preceding Mesquite analyses. Each of the models was temporally stratified over 240 ky, so that dispersal was possible between adjacent areas during warm interglacials (0–10 kya; 90–130 kya; 190–240 kya), but restricted during glacial periods (10–90 kya; 130–190 kya). The models of range evolution were: i) SRM - dispersal from North America through Beringia to Asia; ii) BER - dispersal from Beringia outward to Asia and North America; iii) JAP -dispersal from Japan through Asia and Beringia to North America; iv) CAS - dispersal from Central Asia & Siberia through Beringia to North America; and v) REF - a refugia model that restricted all dispersal prior to 10 kya, but permitted unrestricted dispersal between adjacent regions during the Holocene. The phylogeny and range matrix defining the region from which each specimen was collected were uploaded to the Lagrange configurator [56], biogeographic models were defined as above allowing for two potential ancestral regions per node, and a python file was configured for use in the Lagrange analyses.

### Assessing utility of morphological characters

Stochastic character mapping was used to estimate ancestral states for clades evident in the sect. *Trachyphyllum* phylogeny and provide a framework for investigating the utility of morphological characters for defining evolutionary associations in this section. The characters and traits were derived from the literature and original monographs [15], [17], [19], [21], [22], [25], [57], [58], [59], [60], [61], [62], [63], [64]. Sixteen characters (Table 3) that are widely discussed in the literature and used to delineate taxa within sect. *Trachyphyllum* were coded and then converted to an xml file with Nex2Xml [29] for use in SIMMAP 1.5 [65] to identify potential synapomorphies for each clade, following Calvente et al. [66]. We followed the recommendations of Bollback [29] to estimate priors. First, the overall α and β rate values for each character were sampled in SIMMAP by running MCMC analyses for each character. Then, the best fitting rate values for both α and β were estimated and visualized using the posterior distribution in R [67]. We ran the Ancestral State Reconstruction (ASR) analyses using the α and β rate priors estimated in SIMMAP (Table 3), as well as with 10× and 0.1× priors to check for any prior-based bias in driving the results, following Torices and Anderberg [68]. The ASR was performed for each character, using the post-burn-in trees from MrBayes. To ensure that the analysis was not biased by taxonomic sampling, trees were pruned to one individual per taxon, but we also included one individual from both of the *S. bronchialis* subsp. *austromontana* clades. The outgroups were used for rooting the trees but were excluded from the ASR analyses. Significance for ASR posterior probabilities was determined for well-supported clades in the phylogeny. Ancestral character states that were significant (p>0.95) for a given clade were determined to be useful for inferring evolutionary relationships.

**Table 3:** Morphological characters, SIMMAP priors, and resulting posterior probabilities from the Ancestral State Reconstructions (ASR) for *Saxifraga* sect. *Trachyphyllum*.

Character:		1. Habit (mat)		2. Leaf Shape		3. Leaf Margin		4. Leaf cartilaginous		5. Leaf cilia		6. Leaf apex shape	
Trait:		dense	loose	linear	broad	entire	3-lobed	yes	no	hairy	gland	acute	round
α - prior*:		5.442		3.472		2.825		2.979		5.045		2.866	
β - prior*:		0.240		0.348		0.685		0.440		0.271		0.724	
Clade													
1	Trachyphyllum	0.81	0.19	0.00	**1.00**	**0.99**	0.01	**1.00**	0.00	0.94	0.06	**1.00**	0.00
2		0.46	0.54	0.00	**1.00**	**0.96**	0.04	**1.00**	0.00	0.89	0.11	**1.00**	0.00
3	NA Arctic-Alpine	0.42	0.58	0.00	**1.00**	0.73	0.27	**1.00**	0.00	0.65	0.35	**1.00**	0.00
6		0.82	0.18	0.00	**1.00**	**0.95**	0.05	0.25	0.75	0.71	0.29	**1.00**	0.00
7	Beringia & Asian Is	0.76	0.24	0.00	**1.00**	**1.00**	0.00	**0.98**	0.02	0.70	0.30	**1.00**	0.00
8		0.24	0.76	0.00	**1.00**	**1.00**	0.00	**1.00**	0.00	0.78	0.22	**1.00**	0.00
9		0.42	0.58	0.00	**1.00**	**1.00**	0.00	**1.00**	0.00	0.59	0.41	0.87	0.13
11		0.56	0.44	0.00	**1.00**	**1.00**	0.00	**1.00**	0.00	0.78	0.22	**1.00**	0.00
12	North Pacific	0.22	0.78	0.10	**0.90**	**1.00**	0.00	0.92	0.08	0.82	0.18	**1.00**	0.00
13	Central Asia	0.33	0.67	0.11	0.89	**1.00**	0.00	**1.00**	0.00	0.71	0.29	**1.00**	0.00
14	Spinulosa	0.01	**0.99**	0.00	**1.00**	0.00	**1.00**	0.93	0.07	**0.99**	0.01	0.79	0.21
16	Bronchialis	0.46	0.54	0.00	**1.00**	**0.96**	0.04	**1.00**	0.00	0.89	0.11	**1.00**	0.00
17	Okhotsk	0.42	0.58	0.00	**1.00**	0.73	0.27	**1.00**	0.00	0.65	0.35	**1.00**	0.00
18	Coastal Cascadia	0.08	0.92	0.00	**1.00**	0.82	0.18	**1.00**	0.00	0.88	0.12	**1.00**	0.00

Character:		7. Leaf surface		8. Leaf Keel		9. Stem surface		10. Sepal shape		11. Sepal margin		12. Sepal surface	
Trait:		glab	hairy	none	keeled	glab	gland	triangular	ovate	glab	ciliate	glab	gland
α - prior*:		1.193		2.208		3.070		3.062		2.057		2.473	
β - prior*:		1.259		1.398		0.590		0.497		0.249		0.896	
Clade													
1	Trachyphyllum	**1.00**	0.00	0.02	**0.98**	0.00	**1.00**	0.01	**0.99**	0.01	**0.99**	0.68	0.32
2		**1.00**	0.00	0.00	**1.00**	0.00	**1.00**	0.01	**0.99**	0.00	**1.00**	0.63	0.37
3	NA Arctic-Alpine	**1.00**	0.00	0.00	**1.00**	0.00	**1.00**	0.14	0.86	0.00	**1.00**	0.04	**0.96**
6		**1.00**	0.00	0.59	0.41	0.03	**0.97**	0.00	**1.00**	0.00	**1.00**	**1.00**	0.00
7	Beringia & Asian Is	**1.00**	0.00	0.00	**1.00**	0.00	**1.00**	0.01	**0.99**	0.00	**1.00**	**1.00**	0.00
8		**1.00**	0.00	0.30	0.70	0.02	**0.98**	0.00	**1.00**	0.01	**0.99**	0.94	0.06
9		**1.00**	0.00	0.00	**1.00**	0.00	**1.00**	0.00	**1.00**	0.00	**1.00**	0.74	0.26
11		**1.00**	0.00	0.00	**1.00**	**1.00**	0.00	0.00	**1.00**	0.49	0.51	**1.00**	0.00
12	North Pacific	**1.00**	0.00	**1.00**	0.00	0.92	0.08	0.08	0.92	0.49	0.51	**1.00**	0.00
13	Central Asia	**0.98**	0.02	**1.00**	0.00	0.00	**1.00**	0.08	0.92	0.00	**1.00**	0.05	**0.95**
14	Spinulosa	**1.00**	0.00	0.00	**1.00**	0.05	**0.95**	0.00	**1.00**	**0.99**	0.01	**1.00**	0.00
16	Bronchialis	**1.00**	0.00	0.00	**1.00**	0.00	**1.00**	0.01	**0.99**	0.00	**1.00**	0.63	0.37
17	Okhotsk	**1.00**	0.00	0.00	**1.00**	0.00	**1.00**	0.14	0.86	0.00	**1.00**	0.04	**0.96**
18	Coastal Cascadia	**1.00**	0.00	0.00	**1.00**	0.00	**1.00**	0.00	**1.00**	0.07	0.93	**1.00**	0.00

Character:		13. Petal color		14. Petal spotting				15. Petal length		16. Petal claw			
Trait:		white	yellow	none	yellow	YOR	Purple	< = 4 mm	>4 mm	none	clawed		
α - prior*:		3.774		5.616				2.470		3.666			
β - prior*:		0.247		0.514				0.418		0.567			
Clade													
1	Trachyphyllum	**0.99**	0.01	0.00	0.00	**0.98**	0.02	0.00	**1.00**	**0.99**	0.01		
2		**1.00**	0.00	0.00	0.00	**1.00**	0.00	0.00	**1.00**	**1.00**	0.00		
3	NA Arctic-Alpine	**1.00**	0.00	0.00	0.00	**1.00**	0.00	0.00	**1.00**	**1.00**	0.00		
6		0.86	0.14	0.00	0.01	0.14	0.84	0.00	**1.00**	**1.00**	0.00		
7	Beringia & Asian Is	0.76	0.24	0.00	0.07	0.01	0.92	0.00	**1.00**	**0.96**	0.04		
8		0.85	0.15	0.00	0.00	0.67	0.33	0.00	**1.00**	**1.00**	0.00		
9		0.51	0.49	0.00	0.18	0.81	0.00	0.22	0.78	0.82	0.18		
11		0.54	0.46	0.00	0.00	**0.98**	0.02	0.00	**1.00**	**1.00**	0.00		
12	North Pacific	0.19	0.81	0.00	0.00	0.00	**1.00**	0.08	0.92	**1.00**	0.00		
13	Central Asia	**1.00**	0.00	0.00	0.00	0.59	0.41	0.00	**1.00**	0.87	0.13		
14	Spinulosa	**1.00**	0.00	0.15	0.00	0.83	0.01	0.00	**1.00**	0.94	0.06		
16	Bronchialis	**1.00**	0.00	0.00	0.00	**1.00**	0.00	0.00	**1.00**	**1.00**	0.00		
17	Okhotsk	**1.00**	0.00	0.00	0.00	**1.00**	0.00	0.00	**1.00**	**1.00**	0.00		
18	Coastal Cascadia	**0.99**	0.01	0.00	0.00	**0.96**	0.03	0.00	**1.00**	**1.00**	0.00		

Clades correspond to those in Fig. 5, 7. Posterior probabilities >0.90 are shaded in gray and those >0.95 are in **bold**.

* Priors at 0.10× and 10× were also tested but did not affect significance of posterior probabilities (not shown). Glabrous is abbreviated as glab. Glandular is abbreviated gland. The traits YOR and Purple in 14. Petal spotting stand for yellow-orange-red and pink-purple, respectively.

## Results

We generated a total of 104 new DNA sequences for the Saxifragaceae, 52 for each genetic marker - chloroplast *trn*L-F and nuclear ITS. A visual comparison of the gene trees reconstructed by using *trn*L-F and ITS loci reveals that both markers share the same basic tree topology and are similar to previous published phylogenies [9], [10], [11], [12], [13], but our ITS analyses incorporated many more taxa and yielded higher resolution.

### Circumscription and phylogeny of sect. *Trachyphyllum*

**Figure 3:**
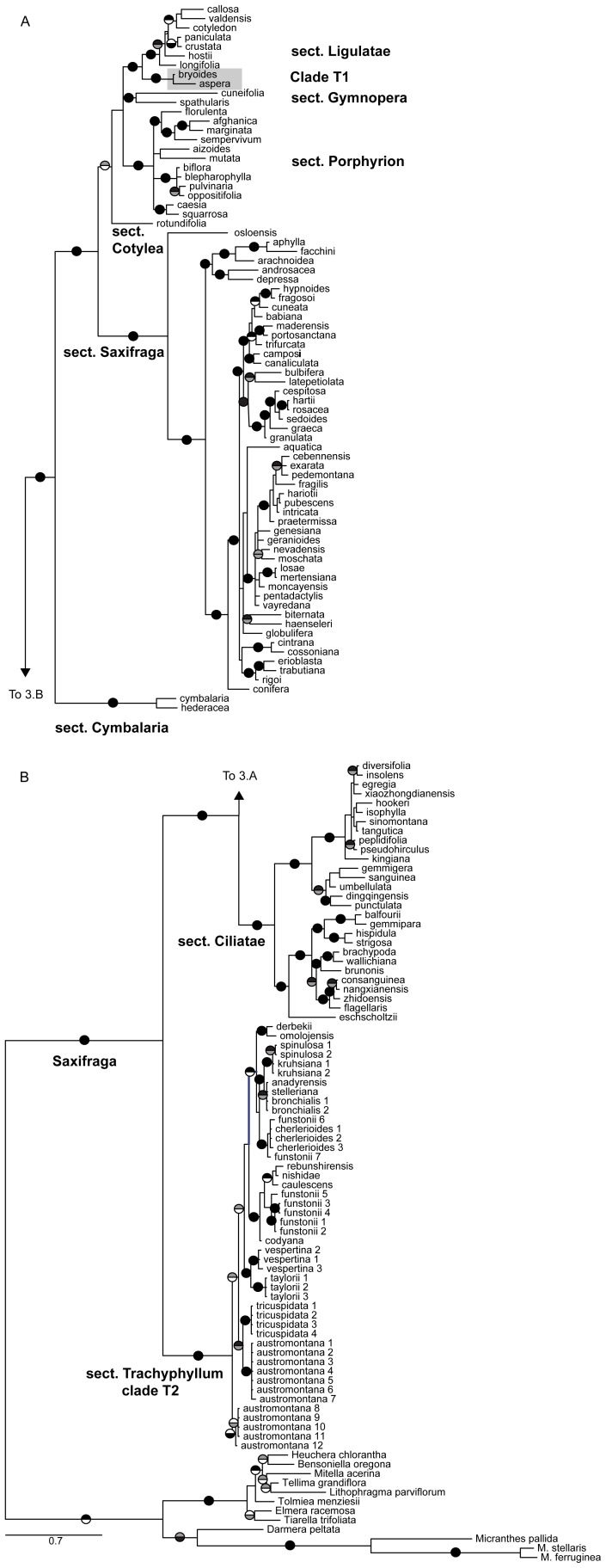
*Saxifraga* phylogeny based on the nuclear ITS regions. Pie graphs on branches indicate relationships that are well-supported under Bayesian (upper; black >0.95, gray 0.90–0.94) and maximum likelihood (lower; black >70, gray 60–69) tree-building methods. The sections of *Saxifraga* are labeled. Clade T1, including both *S. aspera* and *S. bryoides*, is shaded in gray to emphasize the position relative to the main clade of sect. *Trachyphyllum*, Clade T2. For members of the genus *Saxifraga*, only the specific or subspecific epithet is used to label each taxon on the tree.

**Figure 4:**
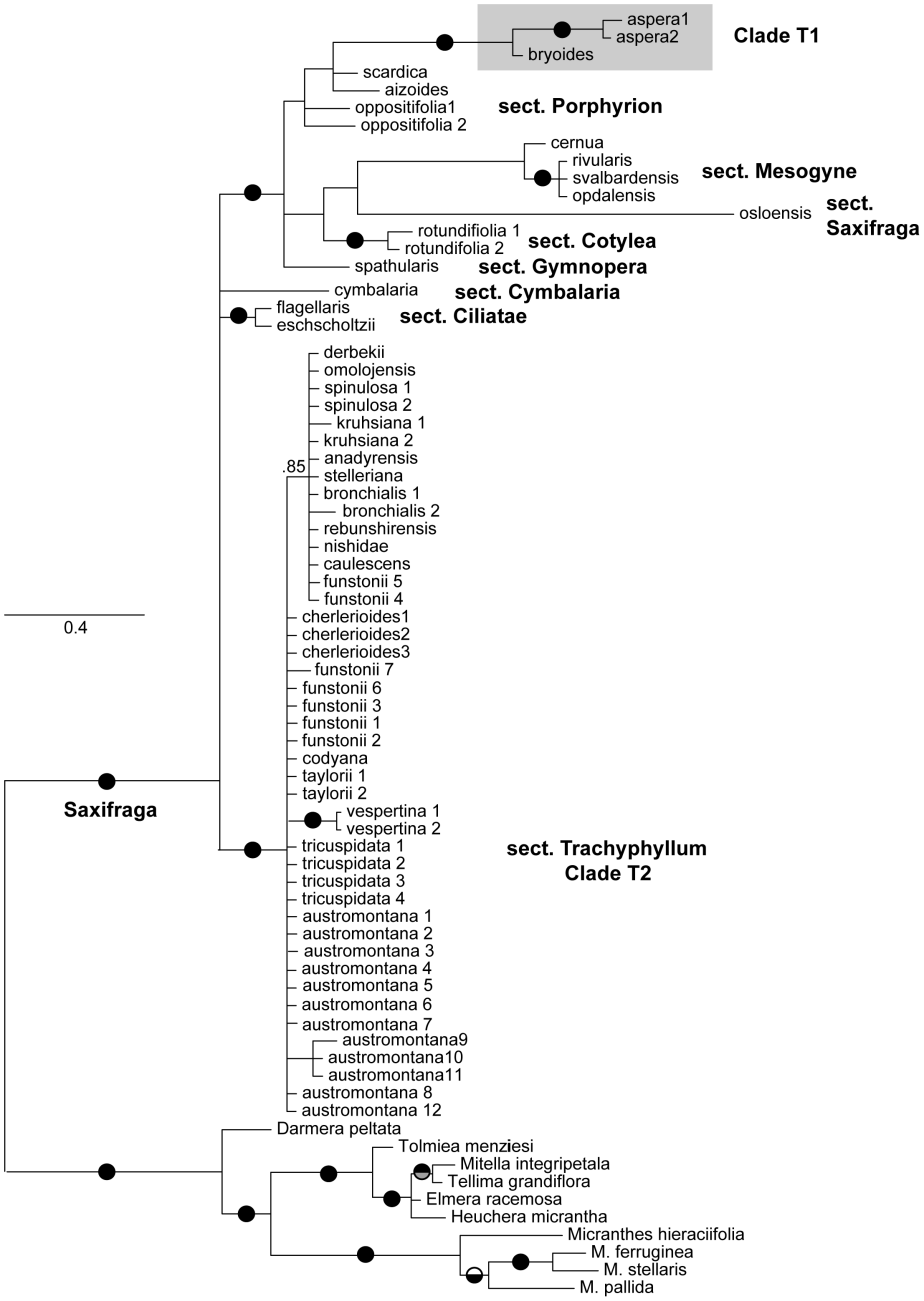
*Saxifraga* phylogeny based on the chloroplast *trn*L-F locus. Pie graphs on branches indicate relationships that are well-supported under Bayesian (upper; black >0.95, gray 0.90–0.94) and maximum likelihood (lower; black >70, gray 60–69) tree-building methods (the low posterior probability of 0.85 for a Eurasia/North America split within sect. *Trachyphyllum* is also given). The sections of *Saxifraga* are labeled. Clade T1, including both *S. aspera* and *S. bryoides*, is shaded in gray to emphasize the position relative to the main clade of sect. *Trachyphyllum*, Clade T2. For members of the genus *Saxifraga*, only the specific or subspecific epithet is used to label each taxon on the tree.

**Figure 5:**
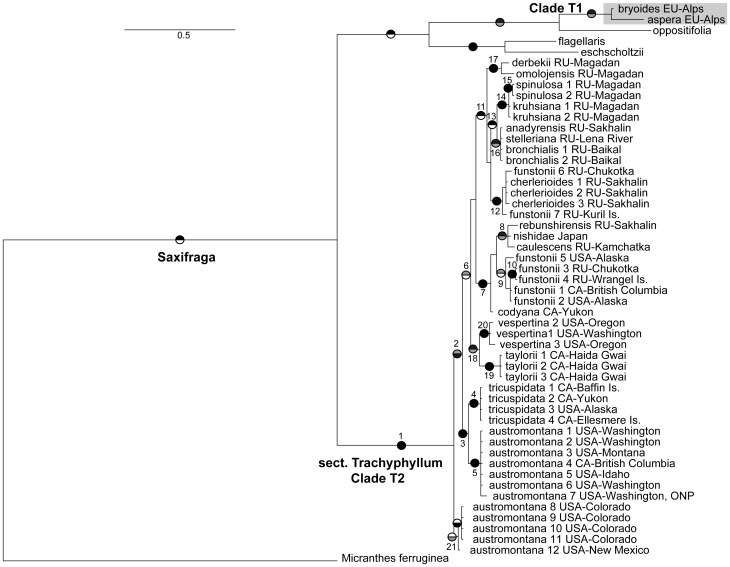
Phylogeny for *Saxifraga* sect. *Trachyphyllum*, based on the entire ITS1, 5.8S, and ITS2 locus data generated in this study. Pie graphs on branches indicate relationships that are well-supported under Bayesian (upper; black >0.95, gray 0.90–0.94) and maximum likelihood (lower; black >70, gray 60–69) tree-building methods. Sub-clades referred to in the text are numbered (1–21). Locations are given for members of sect. *Trachyphyllum*, where CA = Canada, EU = Europe, RU = Russia, USA = United States, Is. = Island, and ONP = Olympic National Park. Clade T1, including both *S. aspera* and *S. bryoides*, is shaded in gray to emphasize the position relative to the main clade of sect. *Trachyphyllum*, Clade T2. For members of the genus *Saxifraga*, only the specific or subspecific epithet is used to label each taxon on the tree.

Both chloroplast and nuclear markers clearly show that sect. *Trachyphyllum* is polyphyletic (Fig. 3, 4, 5), occurring in two separate clades within *Saxifraga*. Clade T1 was comprised of *S. aspera*, the type for the section, and *S. bryoides* and fell within sect. *Ligulatae*. Without those two species, Clade T2 of sect. *Trachyphyllum* is the sister group of the *Saxifraga* clade including the remaining sections and is strictly trans-Beringian. Both markers also advocate a distinction between North American and Asian lineages, though this is not significant for the *trn*L-F region (posterior probability = 0.85). Beyond those findings, the cpDNA tree provided no further resolution within sect. *Trachyphyllum*, and therefore, from here we focus our discussion on the ITS trees. The two ITS phylogenies (Fig. 3 and 5) resolved the same topology with similar support values, though the tree based on the complete ITS dataset generated in this study (Fig. 5) yielded stronger support at a few nodes.

Within sect. *Trachyphyllum* Clade T2, both Bayesian and ML analyses of the two ITS datasets strongly supported several monophyletic groups (sub-clades 3 - North American Arctic-Alpine, 18 - Coastal Cascadia, 7 - Beringia and Asian Islands, 12 - North Pacific, 13 Central Asia [including 14 - Spinulosa and 16 - Bronchialis], and 17 - Okhotsk; Fig. 5), suggesting several taxonomic reversals to prior names: *S. austromontana* Wiegand, *S. funstonii* (Small) Fedde, *S. codyana* Zhmylev (or included within *S. funstonii* at the infraspecific level), and the potential inclusion of *S. stelleriana* Merk. ex. Ser. within *S. bronchialis* (Table 1). As such, we use these classifications in the text from hereon. These sub-clades do not correspond to the previous taxonomic classification schemes (Table 2; sub-sections *Criomorphicae*, *Dentaphyllae*, *Xeromorphicae*) of Khokhryakov [21] and Zhmylev and Khokhryakov [22]. Rather, clade membership appears to be aligned with geographical locations (Table 1, Fig. 5).

### Biogeographic history

Our coalescent-based analyses of population divergence in Mesquite strongly supported scenarios of genetic divergence due to isolation among refugia. The mean DC of the simulated distribution for the models was 8.18 (st. dev. = 2.34). The Refugia (observed DC = 2, p = 0.006) and Southern Rocky Mountain (observed DC = 3, p = 0.028) models could not be rejected at α = 0.05. Eastern origins in Japan (p = 0.879) and Asia (p = 0.339) were rejected. Thus, these tests supported both geographically driven divergence among refugia and a probable origin of sect. *Trachyphyllum* Clade T2 in North America followed by westward dispersal across Beringia and into Asia.

**Figure 6:**
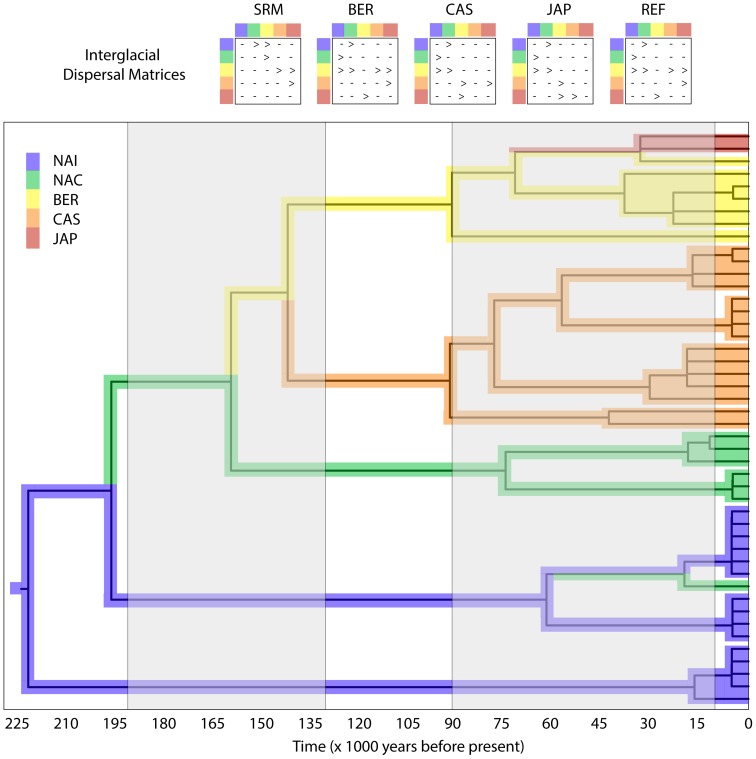
Most likely reconstruction of geographic range evolution in the SRM model for sect. *Trachyphyllum*, inferred through Lagrange. Colors correspond to areas shown on the map in Fig. 1 (blue = North America Interior [NAI]; green = North America Coast [NAC]; yellow = Beringia [BER]; orange = Central Asia & Siberia [CAS]; red = Japan [JAP]). On the phylogeny, white bars represent interglacial periods and gray bars denote glacials. Dispersal matrices for interglacial periods are shown above for each model, with ‘>’ representing directional dispersal and ‘-’ meaning no dispersal. Dispersal was not permitted during glacial periods. The models are as follows: SRM - origin in North America Interior followed by westward dispersal across Beringia and into Asia; BER - dispersal out of Beringia onto both continents; CAS - origin in Central Asia with eastward dispersal across Beringia and into North America; JAP - origin in Japan with eastward dispersal across Beringia and into North America; REF - isolation in each refugium with no dispersal possible until 10 - 0 kya. Time is shown in thousands of years along the bottom of the figure.

The tests of geographic range evolution in Lagrange refined the conclusions from the aforementioned biogeographic tests. The SRM model, with an ancestral range in the interior of North America (NAI) and subsequent westward dispersal across Beringia and into central Asia and south to Japan was the most likely of the hypotheses tested (Table 4). All other models had lower likelihoods, beyond the two log-likelihood unit confidence window (Edwards 1992). The BER model was the next most likely scenario and it also inferred a potential ancestral range in the NAI along with Beringia, though this was not significantly more likely that the other inferred ancestral areas. A closer examination of the range evolution across the phylogeny (Fig. 6) reveals a general pattern of dispersal and cladogenesis - colonists from one area disperse to another and undergo allopatric divergence, with the local extinction of some lineages. Though the models were not constrained by which lineages dispersed to Beringia and then further westward, the resulting inference is one of dispersal primarily along the coastal mountains and islands from Cascadia to Beringia and then independently south to Japan and east to central Asia (Fig. 6).

**Table 4:** Biogeographic inferences from Lagrange.

Model	−ln(L)	Dispersal	Extinction	Ancestral area	−ln(L)
SRM	51.91	79.5	1.943	NAI	52.55
BER	54.73	71.29	2.013	NAI/BER	56.13
				NAC	56.15
				BER	56.56
				BER/NAC	57.88
JAP	78.14	100	3.689	BER	78.57
				BER/JAP	79.80
				JAP	80.92
CAS	83.09	100	3.811	BER	83.53
				BER/CAS	84.76
				CAS	85.85
REF	96.21	100	3.707	BER	96.21

All Lagrange [28] models permitted dispersal during interglacials (0–10 ky; 90–130 ky; 190–240 ky) between any adjacent geographic areas as follows (Fig. 6): SRM from North America through Beringia to Asia; BER from Beringia outward; JAP from Japan through Beringia to North America; CAS from Central Asia & Siberia through Beringia to North America; except for REF, which restricted all dispersal prior to 10 kya. For all other models, dispersal was restricted during glacial periods (10–90 ky; 130–190 ky). The root age was estimated at 220 kya, based on 1.72 substitutions/site/year [55]. All ancestral areas inferred for the root node of each model are shown.

### Morphology

**Figure 7:**
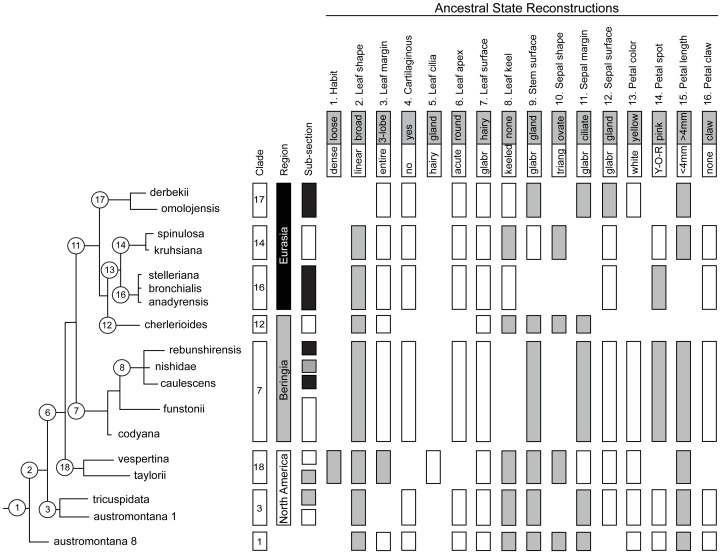
Ancestral State Reconstructions (ASR) for the sub-clades of *Saxifraga* sect. *Trachyphyllum*. Only members of sect. *Trachyphyllum* Clade T2 are shown (not *S. aspera* or *S. bryoides* because they were not included in the morphological analyses given their divergent phylogenetic positions). Sub-clades used in the ASR analyses are numbered (from Fig. 5). The first three columns denote the focal clades depicted in the subsequent bar plots (note that the bottom row is for sub-clade 1 = *Trachyphyllum*), geographic location (white = North America, gray = Beringia, black = Eurasia), and subsection classification (white = *Criomorphicae*, gray = *Dentaphyllae*, black = *Xeromorphicae*), respectively. These columns are followed by the morphological characters and traits inferred for each sub-clade through the ASR analyses in SIMMAP. Only significant ASR's are shown; the absence of a bar indicates no significant trait was inferred. White and gray shading denote traits within each character as labeled.

Our analyses into the morphological traits that define sub-clades in sect. *Trachyphyllum* Clade T2 demonstrated that while species-specific traits are useful in delineating taxa, few characters are phylogenetically informative and many have been misleading in the classification of sub-sections and series (Table 3, Fig. 7). The overall, statistically-significant (at posterior probability >0.95) ancestral state for Clade T2 included broad, non-cartilaginous, un-keeled leaves with entire margins, acute apices, and glabrous surfaces; glandular hairs on stems; ovate sepals with ciliate margins; and white, un-clawed, petals, that are >4 mm with yellow-orange-red spotting.

The seven well-supported sub-clades differed by exhibiting the following traits (Table 3, Fig. 7). The North American Arctic-Alpine sub-clade is marked by broad, non-cartilaginous, un-keeled, glabrous leaves with acute tips; glandular stem surfaces; sepals with ciliate margins and glandular surfaces; white, un-clawed petals that have yellow-orange-red spots and are >4 mm long. Also in North America, the Coastal Cascadia sub-clade forms loose mats and has broad, 3-lobed, un-keeled, glabrous leaves with non-glandular cilia; glandular stem surfaces; ovate, glabrous sepals; and white petals >4 mm long. The Beringia and Asian Islands sub-clade exhibits broad, non-cartilaginous, un-keeled leaves with entire margins, acute tips, and glabrous surfaces; glandular stem surfaces; glabrous sepals with ciliate margins; and white, un-clawed petals that are >4 mm long and have pink-purple spotting. The North Pacific taxa fit the sect. *Trachyphyllum* ASR without the acute leaf apex or any inferences on petal morphology. Members of the Central Asian sub-clade, which was further subdivided into the Spinulosa and Bronchialis clades, share the characteristics of broad, non-cartilaginous leaves with entire margins, acute tips, and glabrous surfaces; ovate, glabrous sepals; and un-clawed petals that are >4 mm long. Finally, the Okhotsk sub-clade displayed non-cartilaginous, glabrous, keeled leaves with entire margins and an acute apex; glandular stem surfaces; glandular sepals with cilia, and white petals >4 mm long. Sub-clades showed a tendency for several other traits at p = 0.90 (Table 3).

## Discussion

### Evolutionary relationships of sections within *Saxifraga*

Our analyses corroborated previous findings for *Saxifraga*, including monophyly of the genus and strong support for multiple clades/sections of *Saxifraga* from both chloroplast and nuclear markers. Given that the analysis of *Saxifraga* on the whole was not the focus of this study, we will only comment briefly on aspects of the genus tree that are not related to sect. *Trachyphyllum*. Seven other monophyletic clades are evident in the genus tree, with sect. *Ciliatae* being the most closely related to sect. *Trachyphyllum* and one of the basal-most clades [58]. Our sequence data for *S. eschscholtzii* placed it within sect. *Ciliatae* as anticipated [15], but in contrast to the earlier works of Losina Losinskaya [25] who had included it within sect. *Trachyphyllum*. Many of the previously un-analyzed GenBank accessions of Asian *Saxifraga* were also placed within sect. *Ciliatae*. *Saxifraga* sect. *Saxifraga* is a well-supported clade as had been previously inferred by Vargas [13], with *S. osloensis* appearing basal as in Zhang et al. [11]. The remaining sections are strongly supported (*Cotylea* Tausch, *Porphyrion* Tausch, *Ligulatae*, and *Gymnopera* D. Don). The only major differences within *Saxifraga* warranting taxonomic revision are concerning the circumscription of sect. *Trachyphyllum.*

### Revised circumscription of sect. *Trachyphyllum*

The division of sect. *Trachyphyllum* into two separate groups, Clade T1 (*S. aspera* and *S. bryoides*) and Clade T2 (all others) has huge implications for clarifying the evolutionary history of the section (see DeChaine [69] for a formal revision). This finding fits with Zhmylev and Khokhryakov's [22] suggestion that subsection *Fibrosophyllae* should be considered a section of its own given the subsection's geographic and morphological uniqueness [22]. Indeed, *S. aspera* and *S. bryoides* are the only taxa to have been included within sect. *Trachyphyllum* that occur in the mountains (Alps, Apennines, Carpathians, and Pyrenees) of Europe, outside of Russia. They are also distinct environmentally for their reliance on silicaceous substrates. From a morphological standpoint, they differ from the other taxa in having a large, yellow blotch at the base of their white petals, leaves with fibrous margins and forward directed cilia, and conspicuous buds in the leaf axils at flowering time.

Taxa previously ascribed to sect. *Trachyphyllum* in the subsections *Criomorphicae*, *Xeromorphicae*, and *Dentaphyllae* form the monophyletic Clade T2. That said, the sub-sections and associated series [21], [22] do not reflect evolutionary entities given the phylogeny inferred from our molecular markers, but rather appear to be largely based on convergent and/or environmentally affected characters. The seven narrowest, well-supported sub-clades in Clade T2 (Fig. 5, 7) are as follows, using the taxonomic ranks suggested by the phylogeny (Table 1): i) North American Arctic-Alpine (*S. austromontana* and *S. tricuspidata*), ii) Coastal Cascadia (*S. taylorii* and *S. vespertina*), iii) Beringia and Asian Islands (*S. funstonii* and *S. codyana*, and a well-supported sub-clade with *S. nishidae*, *S. rebunshirensis*, and *S. caulescens*), iv) North Pacific (*S. cherlerioides*), v) Okhotsk (*S. derbekii* Sipliv. and *S. omolojensis* A. P. Khokhr.), and Central Asia including vi) Spinulosa (*S. spinulosa* and *S. kruhsiana* Fisch. ex Ser.), and vii) Bronchialis (*S. bronchialis* [including *S. stelleriana*] and *S. bronchialis* subsp. *anadyrensis* (Losinsk.) Kozhern).

### Sub-clades within *Trachyphyllum* Clade T2

#### North American Arctic-Alpine (sub-clade 3)

The basal-most lineages of *Trachyphyllum* Clade T2 belong to the North American *S. austromontana* and *S. tricuspidata*. *Saxifraga austromontana* is a North American endemic, from south of the Pleistocene ice sheets. *Saxifraga tricuspidata* is restricted to the northern part of the continent and neighboring Greenland. These two taxa are morphologically and molecularly distinct, though the genetic divergence between them is not great, suggesting a recent split and given the distributions of the two taxa, primarily north and south of the Laurentide Ice Sheet, the division was likely mediated by a glacial period. Interestingly, other than Wiegand [61] and Small [57], *S. austromontana* has always been assumed to be a subspecies of *S. bronchialis*. And, *S. tricuspidata* has traditionally been thought of as unique within the section, ‘clearly distinct from North American and most Asian species’ [22], given its 3-lobed leaves. Haworth [70] even placed it in a different genus (*Leptasea* Haw. rather than *Ciliaria* Haw.) than the other members of sect. *Trachyphyllum* and Don [71] considered it to be in a different section. That said, one described form of *S. tricuspidata*, f. *subintegrifolia* (Abrom.) Polunin, has entire leaves, similar to that of *S. austromontana*, though the two species are still readily distinguished by the presence of glandular hairs on the leaf margins of *S. tricuspidata*. Consequently, Calder and Savile [19] questioned whether the two were distinct species that had recently met during post-Last Glacial Maximum (LGM) range expansions. Indeed, they are closely related and geography appears to be the guide to the evolutionary history of these two. The ASR analysis revealed several traits that the two taxa share, though none differed from that of the section. This is likely because the two differ markedly in vegetative characters (such as entire vs. 3-lobed leaf margins). On a final note concerning these taxa, the southernmost populations of *S. austromontana* from Colorado and New Mexico fell outside the North American Arctic-Alpine sub-clade, though only well-supported in the ML analyses, hinting at a Southern Rocky Mountain origin for the section. Given its basal position, the this sub-clade holds keys to the origin and process of speciation in the group.

#### Coastal Cascadia (sub-clade 18)

The Cascadian sub-clade includes two distinct species endemic to the Pacific Northwest: *S. vespertina* of Oregon and Washington (USA) and *S. taylorii* of Haida Gwaii, British Columbia (Canada). Both species exhibit very patchy, narrow distributions where micro-environments provide suitable habitat. These two taxa were hypothesized to be close relatives of one another [19], though early molecular work suggested that they were more divergent than previously thought [72]. Our analyses show that indeed they are closely related species, but, the *trn*L-F sequences of *S. vespertina* are quite different from *S. taylorii* (Fig. 4). The two species share many characters in common that distinguish them from other clades in the section, most noticeably the presence of 3-lobed leaves (though this trait is variable in *S. vespertina*) that are soft to the touch. Yet, the flowers differ between the two, given the lack of spotting and larger petals in *S. taylorii*. These two species likely resided in coastal glacial refugia, splitting only recently due to that isolation. Thus, divergence and endemism in these North American taxa is probably a product of allopatry, mediated by differing environmental selection pressures. Yet, their evolutionary relationship to the rest of the section, and thus the history of these species, remains unclear - Do they have a tighter association with North American or Asian taxa? One taxon that could provide a clue to this is *S. arinae* of Russia, which Zhmylev [14] hypothesized to be related to *S. vespertina* (but as stated previously, this species is likely synonymous with *S. nishidae*). Unfortunately, we were unable to obtain specimens of *S. arinae* for this study, but *S. nishidae* is clearly genetically distinct (Fig. 3, 5).

#### Beringia and Asian Islands (sub-clade 7)

The constituent taxa, *S. funstonii*, *S. codyana*, *S. rebunshirensis*, *S. nishidae*, and *S. caulescens*, are so morphologically diverse that they include members of each of the three subsections described by Khokhryakov [21] and Zhmylev and Khokhryakov [22], assuming *S. nishidae* were to fall within the *Dentaphyllae* as it should given its tricuspid leaves. All evidence suggests that these taxa are narrow endemics, with *S. funstonii* being the most widely distributed, from Alaska to Chukotka and Wrangel Is. of Russia. *Saxifraga funstonii* has strong support for monophyly (clade 9), excluding *S. codyana*. The question as to whether *S. codyana* is a species [73] or not [18], [74] still remains. At the minimum, this taxon should not be associated with *S. bronchialis*. A southern sub-clade (8), including *S. nishidae*, *S. rebunshirensis*, and *S. caulescens*, ranges from Kamchatka to Japan. McGregor [16] doubted that *S. caulescens* was anything more than a local form of *S. bronchialis*, but we show here that it is distinct. Given the morphological diversity of this small group, the ASR analysis was similar to that of the section overall, though petal spotting was inferred to be pink-purple. This group highlights the importance of Beringia and the mountains of Japan in preserving lineages and producing endemic taxa.

*North Pacific (sub-clade 12)* - The accessions falling into the North Pacific sub-clade include both taxa determined to be *S. cherlerioides* and *S. funstonii*, ranging geographically along eastern Russia from Chukotka, through Sakhalin, and the Kuril Islands and across the Aleutians into southern Alaska and the Yukon. Though these two ‘species’ were viewed as being very similar morphologically and closely related evolutionarily [75], *S. funstonii* of the Beringia and Asian Islands sub-clade is very distinct from a molecular standpoint. Morphologically they differ in that, unlike *S. funstonii*, *S. cherlerioides* has un-clawed petals, stamens shorter than petals, and smaller flowers and capsules. Yet, given the morphological similarity (and historic confusion) between *S. cherlerioides* and *S. funstonii*, it is not unlikely that the two *S. funstonii* specimens were mis-identified. The presence of two phylogenetically distinct trans-Beringian sub-clades further highlights that region's role in intercontinental dispersal and diversification.

#### Okhotsk (sub-clade 17)

*Saxifraga derbekii* and *S. omolojensis* are morphologically distinct species that had not previously been hypothesized to be closely related, beyond both being suspected to nest with *S. bronchialis* in the *Xeromorphicae* subsection [22]. Yet, the two species form a well-supported clade. Again, geography holds clues to their association in that both species occur in Magadan, Russia, near the Sea of Okhotsk and most likely are endemic species of that region. *Saxifraga derbekii* is known for its pale pinkish spots at the base of its white petals and linear-lanceolate leaves, while *S. omolojensis* has yellow and red spots and clawed petals and pubescent, awl-shaped leaves. The characters inferred through ASR that unite them and distinguish them from most other sub-clades in the section are keeled leaves and glandular sepal surfaces.

#### Central Asia (sub-clade 13): Spinulosa and Bronchialis

Much of the diversity of sect. *Trachyphyllum* has been characterized as belonging to two aggregates (sub-sections): Spinulosa (*Criomorphicae*) and Bronchialis (*Xeromorphicae*), but our analyses show that both these groups are polymorphic and that these two species are relatively closely related. The ASR for the Central Asia sub-clade did not differ from that of sect. *Trachyphyllum* on the whole, but there are some distinguishing traits between taxa. The Spinulosa aggregate (sub-clade 14) has been reduced by our analyses to include only *S. spinulosa* and *S. kruhsiana.* The two specimens of *S. spinulosa* are monophyletic, though the same is not true for *S. kruhsiana*. This, and the short branch lengths of this clade suggest that the two taxa are potentially either closely related species or subspecies. These taxa range across Russia, from the Urals to Kamchatka, Chukotka, and Okhotsk. The Bronchialis sub-clade (16) consists of *S. bronchialis*, *S. stelleriana* and *S. bronchialis* subsp. *anadyrensis*, which are all likely subspecies or varieties of *S. bronchialis*. All three taxa had been previously hypothesized to be in the same subsection (*Xeromorphicae*), but with several other taxa as well. While our findings support the classification of *S. bronchialis* subsp. *anadyrensis*, our inferences suggest that *S. stelleriana* may not deserve any taxonomic distinction as noted by Elven et al. [17]. This sub-clade is geographically wide-ranging in Russia, from the Lena River and Lake Baikal to Sakhalin. The ASR inferences differed between the two groups in the character states for leaf keel and petal spotting. These findings have helped to clarify the number of endemic species and infraspecies that have been included within both *S. spinulosa* and *S. bronchialis*.

### Geographic drivers of divergence

From the preceding discussion, the tight association between phylogenetic sub-clades and geographic regions should be apparent. Indeed, with the exclusion of *S. aspera* and *S. bryoides* (Clade T1), the geographic distribution of sect. *Trachyphyllum* is limited to the arctic, alpine, and subalpine environs on either side of the north Pacific and in Beringia. Both of our approaches for testing biogeographic models, coalescent simulations under models of population divergence and maximum likelihood tests of geographic range evolution, strongly pointed to an origin of the section in the interior of North America followed by dispersal and divergence across Beringia and into Asia. Moreover, our analyses suggested an important role of coastal areas in facilitating westward dispersal around the north Pacific Rim, confirming the suspicions of Calder and Savile [19] of an enhanced northward coastwise rate of spread within sect. *Trachyphyllum* of North America. This goes against the more commonly inferred eastward migration of animals across the Bering Land Bridge [76], but fits with what has been found for other arctic plants [77] and underscores the importance of southern mountain refugia in the evolution of the arctic flora [8].

Along the range expansion route, colonization of new areas coincided with divergence of lineages and allopatric speciation. Those divergence events line up with refugial isolation during glacial periods (Fig. 6), but given that the estimates of divergence times in our gene tree are based on a very rough estimate of substitution rate [55] we feel that the temporal relationship should be viewed with caution. As lineages dispersed around the north Pacific Rim, several refugia lying in this arc could have been safe harbors that promoted divergence within sect. *Trachyphyllum* during glacial times. Traditionally, Beringia has been viewed as the major refugium for arctic plants in the region [78], and its role in the maintenance, dispersal, and potential diversification of lineages is evident herein. But, other putative refugia south of the ice in western North America (the Southern Rocky Mountain Refugium, Klamath region, Haida Gwaii [79], [80], [81]) and eastern Asia (the mountains of Japan and neighboring islands [82], [83], [84]) and west of Beringia (Central Asia [85], [86]) had a clear impact on the origin and evolutionary history of this section. In addition, north-south splits were observed for several sub-clades in the phylogeny (*S. taylorii* and *S. vespertina*; *S*. *austromontana* and *S. tricuspidata*; *S. funstonii* and *S. nishidae/S. rebunshirensis/S. caulescens*), suggesting repeated rounds of isolation and divergence that fit with the pattern of glacial-interglacial cycles of range expansion and fragmentation. By uncovering these patterns, our analyses have begun to address some of the questions regarding the origin of the trans-Pacific tundra and taiga flora [78], [87], [88], [89].

### The utility of morphological characters in estimating evolutionary relationships

Our analyses of the utility of morphological characters in defining evolutionary relationships in sect. *Trachyphyllum* suggest that several traits are useful for identifying an individual taxon and many are either evolutionarily convergent or vary in response to environmental conditions. Some of the traits were taxon specific, such as the lack of spotting in *S*. *taylorii* or the hairiness of the leaf surfaces in *S. omolojensis*, and thus not useful for inferring relationships among species. Other traits appeared multiple times across the tree, for example the 3-lobed leaves of S. *tricuspidata*, *S. taylorii*, *S. vespertina*, and *S. nishidae*, suggesting multiple origins. Several traits departed from the overall ASR for sect. *Trachyphyllum*, and thus appeared useful for determining clade membership at α = 0.05, but also overlap in several clades (i.e., leaf keel, sepal surface, petal spotting). Thus, our ASR analyses suggest that morphology alone is not a good means of determining evolutionary relationships in sect. *Trachyphyllum*.

Ours is not the first study to address issues concerning which morphological characters are useful in the classification of the *Saxifraga*. Webb and Gornall [15] summarized the characterization of the flowers of *Saxifraga* as usually having 5 sepals, 5 petals, 10 stamens in 2 whorls (outer opposite sepals and inner opposite petals), 2 carpels that are united at least in part, and an absence of a free hypanthium. They further noted that the highly variable vegetative characters used to separate the sections, subsections, and series are often correlated with habitat and that the more useful characters were associated with the hairs, leaf crystals, presence of hydathodes, ovary position, and petal coloring. Our analyses corroborate their assertions for sect. *Trachyphyllum* as well. For sect. *Saxifraga*, Vargas [13] showed that morphological characters could not be mapped onto the phylogenetic clades, as with our findings for sect. *Trachyphyllum*. Vargas [13] suggested that the incongruence could be due to convergence of traits, lineage sorting, or hybridization and reticulation, citing the greatest support for the latter. Indeed, interspecific hybrids are common in *Saxifraga* (mostly notably in sect. *Saxifraga*, sect. *Gymnopera*, sect. *Ligulatae*, and sect. *Porphyrion*), which has made classifications based on morphology difficult [15], [16]. The potentially recent splits observed between sub-clades in sect. *Trachyphyllum*, the repeated independent origin of some traits (e.g., 3-lobed leaves), and the strong possibility of hybridization suggest that all three factors could be diminishing the utility of morphological characters for estimating evolutionary relationships.

### Hybridization and the evolution of sect. *Trachyphyllum*

Though our study did not directly investigate hybridization within sect. *Trachyphyllum*, the phenomenon has probably exacerbated the taxonomic uncertainty [14] and played a significant role in the history of the section [20]. Among the Eurasian species, many transitional forms between *S. bronchialis* and *S. spinulosa* have been documented that probably represent hybridization events [15], [16], [17], [20], [25]. Indeed, Elven et al. [17] suggested that because of the putative hybridization between *S. bronchialis* and *S. spinulosa*, the two aggregates/sub-sections (*Xeromorphicae* and *Criomorphicae*) were probably more closely related than previously suspected. This hypothesis was borne out in our phylogeny where the two were placed within the CAS sub-clade (Fig. 5,7). Though not restricted to that sub-clade, the diploid nature of the taxa could have facilitated hybridization [17], [90]. The evolutionary implications of those hybridization events remain uncertain, but for one hypothetical hybrid, our molecular analyses have helped to resolve the history. Siplivinsky [20] postulated that *S. caulescens* was “undoubtedly” a hybrid of *S. bronchialis* and *S. spinulosa*, but if that were the case *S. caulescens* would have been nested with them in the CAS sub-clade rather than associated with the Japanese taxa in the BER sub-clade. Other Eurasian species in our analyses (e.g., *S. derbekii* and *S. omolojensis*) are phylogenetically distinct and exhibit unique, rather than intermediate, morphologies implying that they did not arise through hybridization [91], [92]. Furthermore, there is nothing to suggest hybridization among the North American species of sect. *Trachyphyllum* [15]. Calder and Savile [19] clearly argued that there was no evidence for hybridization between *S. austromontana* and *S. tricuspidata*, *S. funstonii* and *S. tricuspidata*, or *S. funstonii* and *S. cherlerioides* (disputing an earlier suggestion of Losina-Losinskaya [25]) and Goertzen [71] experimentally showed that *S. taylorii* and *S. vespertina* would not hybridize. Finally, there is no indication of hybridization between the European species, *S. aspera* and *S. bryoides* [15] (Clade T1). Yet, hybridization has served as an important evolutionary mechanism for some groups of the Saxifragaceae (reviewed in [9]), and in particular *Saxifraga* [13], [15], [16], begging further analyses that employ a suite of genetic loci [90] to clarify the extent that hybridization has impacted the evolutionary history of sect. *Trachyphyllum*.

## Conclusions

We view this study as a first step in elucidating the evolutionary history of *Saxifraga* sect. *Trachyphyllum*, while much work remains to be done. Our molecular analyses have set up hypotheses, providing a new foundation for the circumscription of this section, uncovering the separate placement of *S. aspera* and *S. bryoides*, inferring a basal position of sect. *Trachyphyllum* within *Saxifraga*, suggesting a middle-ground for the number of species within the section, emphasizing the role of refugia in evolution and diversification, and revealing the usefulness of various morphological characters in categorizing members of the section. From a taxonomic perspective, we have added to our understanding of the diversity within *Saxifraga* and revealed the importance of sect. *Trachyphyllum* as a sister clade to the remainder of the genus. Our analyses suggest the following taxonomic classifications: *S. austromontana* Wiegand, *S. funstonii* (Small) Fedde, possibly *S. codyana* Zhmylev, and the potential inclusion of *S. stelleriana* Merk. ex. Ser. within *S. bronchialis* L. Major questions still remain regarding i) the position of the Coastal Cascadia sub-clade (*S. taylorii* and *S. vespertina*), ii) the diversity within the Beringia and Asian Islands sub-clade (particularly that of *S. nishidae*, *S. rebunshirensis*, and S. *caulescens*), and iii) the classification of members of sect. *Trachyphyllum* that were unavailable for this study. In terms of geography, our study underscored the important roles of southern mountains and westward dispersal across refugia in promoting diversification within this group. Furthermore the arctic-alpine environment and the trans-Beringia region were highlighted as critical factors in the evolution of *Saxifraga*. But, more thorough tests incorporating numerous samples per species and across their ranges will help to resolve the geographic origin and dispersal routes in the region. The morphological assessment identified useful and convergent characters that have been used in classifying members of sect. *Trachyphyllum* and *Saxifraga* in general. A thorough morphological examination of all sect. *Trachyphyllum* taxa remains to be done and should be performed on fresh specimens in order to view characters that may change with drying (e.g., petal color and spotting). Future comparative analyses within this section, incorporating many individuals and numerous nuclear loci, will likely permit a deeper understanding of how evolutionary processes have impacted speciation in *Saxifraga*, the arctic-alpine flora, and the biogeographic history of the north Pacific.

## Supporting Information

Appendix S1 GenBank accession numbers for data not generated in this study. Data are labeled as follows: Chloroplast = C, ITS = I, ITS1 = I1, ITS2 = I2.
(DOC)
