# Supplementary material for: On the Evolutionary and Biogeographic History of Saxifraga sect. Trachyphyllum (Gaud.) Koch (Saxifragaceae Juss.)
Source: PLoS One. 2013 Jul 26;8(7):e69814. doi: 10.1371/journal.pone.0069814 (PMC3724901; doi:10.1371/journal.pone.0069814)
Supplement: Appendix S1 — GenBank accession numbers for data not generated in this study. Data are labeled as follows: Chloroplast = C, ITS = I, ITS1 = I1, ITS2 = I2. (DOC) [file pone.0069814.s001.doc]

Outgroups: Bensoniella oregona (I- AF158953), Darmera peltata (C- AF374795, I- AB292041), Elmera racemosa (C- AF374804, I- AB248849), Heuchera chlorantha (I- AF158955), Heuchera micrantha (C- AF374806), Lithophragma parviflorum (I- AF158942), Micranthes pallida (C- JN02274, I- EU158863), Micranthes stellaris (C- AF374802, I1- AF374827, I2- AF374828), Mitella acerina (I- AF158958), Mitella integripetala (C- AB116716), Tellima grandiflora (C- AF374805, I- AF158952), Tiarella trifoliata (I- AB248856), Tolmiea menziesii (C- AF374803, I- AB248857).

Saxifraga: afghanica (I- EU158853), aizoides (C- AF374787, I- AF504547), androsacea (I- AF261159), aphylla (I- AF261160), aquatica (I- AF261161), arachnoidea (I- AJ233859), babiana (I- AJ233879), balfourii (I- JN102235), biflora (I- AF504546), biternata (I- AF261163), blepharophylla (I- AF504545), brachypoda (I- EU158855), brunonis (I- JN102236), bulbifera (I- AF261166), caesia (I1- AF087588, I2- AF087618), callosa (I1- AF08758, I2- AF087611), camposii (I- AJ233863), canaliculata (I- AF261167), cebennensis (I- AF261169), cernua (C- AF374779), cespitosa (I- AF261170), cintrana (I- AF261171), conifera (I- AJ233864), consanguinea (I- EU158837), cossoniana (I- AF261172), cotyledon (I1- AF087584, I2- AF087614), crustata (I1- AF087583, I2- AF087613), cuneata (I- AJ233880), cuneifolia (I1- AF087595, I2- AF087625), cymbalaria (C- AF374777, I1- AF087599, I2- AF087629), depressa (I- AF261173), dingqingensis (I- EU158857), diversifolia (I- JN102241), egregia (I- EU158836), erioblasta (I- AJ233866), exarata (I- AJ233861), facchini (I- AF261175), florulenta (I1- AF087591, I2- AF087621), fragilis (I- AF261176), fragosoi (I- AJ233867), gemmigera (I- EU158856), gemmipara (I- EU158859), genesiana (I- AF261178), geranioides (I- AF261192), globulifera (I- AJ233868), graeca (I- AF261179), granulata (I- AJ233860), haenseleri (I- AF261180), hariotii (I- AF261181), hartii (I- AF261189), hederacea (I- AF261182), hispidula (I- JN102237), hookeri (I- EU158840), hostii (I1- AF087579, I2- AF087609), hypnoides (I- AJ233875), insolens (I- EU158841), intricata (I- AJ133030), isophylla (I- EU158848), kingiana (I- EU158851), latepetiolata (I- AF261183), longifolia (I1- AF087585, I2- AF087615), losae (I- AJ133027), maderensis (I- AJ233882), marginata (I1- AF087589, I2- AF087619), mertensiana (I- AY231367), moncayensis (I- AJ133028), moschata (I- AF261184), mutata (I1- AF087593, I2- AF087623), nangxianensis (I- EU158850), nevadensis (I- AF261185), opdalensis (C- AJ238769), oppositifolia2 (C- AF374782), osloensis (C- AF374788, I1- AF087608, I2- AF087638), paniculata (I1- AF087586, I2- AF087616), pedemontana (I1- AF087606, I2- AF087636), pentadactylis (I- AJ133031), peplidifolia (I- EU158843), portosanctana (I- AJ233883), praetermissa (I- AF261186), pseudohirculus (I- EU158844), pubescens (I1- AF261187, I2- AF261188), pulvinaria (I- EU158860), punctulata (I- EU158854), rigoi (I- AJ233878), rivularis (C- GU30253), rosacea (I- AF261190), rotundifolia (C- AF374783, I1- AF087598, I2- AF087628), rotundifolia2 (C- X7989), sanguinea (I- EU158849), scardica (C- AF374784), sedoides (I- AF261191), sempervivum (I1- AF087590, I2- AF087620), sinomontana (I- EU158834), spathularis (C- AF374785, I- AJ233858), squarrosa (I1- AF087587, I2- AF087617), strigosa (I- EF369514), svalbardensis (C- AJ238771), tangutica (I- EU158858), trabutiana (I- AF482693), trifurcata (I- AJ233885), umbellulata (I- EU158833), valdensis (I1- AF087582, I2- AF087612), vayredana (I- AJ133029), wallichiana (I- U158847), xiaozhongdianensis (I- EU158835), zhidoensis (I- EU158862).
